# Biomechanical analysis of prey capture in the carnivorous Southern bladderwort (*Utricularia australis*)

**DOI:** 10.1038/s41598-017-01954-3

**Published:** 2017-05-11

**Authors:** Simon Poppinga, Lars Erik Daber, Anna Sofia Westermeier, Sebastian Kruppert, Martin Horstmann, Ralph Tollrian, Thomas Speck

**Affiliations:** 1 Plant Biomechanics Group, Botanic Garden, University of Freiburg, Schänzlestraße 1, D-79104 Freiburg im Breisgau, Germany; 2 Freiburg Centre for Interactive Materials and Bioinspired Technologies (FIT), Georges-Koehler-Allee 105, D-79110 Freiburg im Breisgau, Germany; 3 Department of Animal Ecology, Evolution and Biodiversity, Ruhr-University Bochum, Universitätsstraße 150, D-44780 Bochum, Germany

## Abstract

We recorded capture events (CEs) of the daphniid *Ceriodaphnia dubia* by the carnivorous Southern bladderwort with suction traps (*Utricularia australis*). Independent to orientation and behavior during trap triggering, the animals were successfully captured within 9 ms on average and sucked in with velocities of up to 4 m/s and accelerations of up to 2800 *g*. Phases of very high acceleration during onsets of suction were immediately followed by phases of similarly high deceleration (max.: −1900 *g*) inside the bladders, leading to immobilization of the prey which then dies. We found that traps perform a ‘forward strike’ during suction and that almost completely air-filled traps are still able to perform suction. The trigger hairs on the trapdoors can undergo strong bending deformation, which we interpret to be a safety feature to prevent fracture. Our results highlight the elaborate nature of the *Utricularia* suction traps which are functionally resilient and leave prey animals virtually no chance to escape.

## Introduction

**Figure 1:**
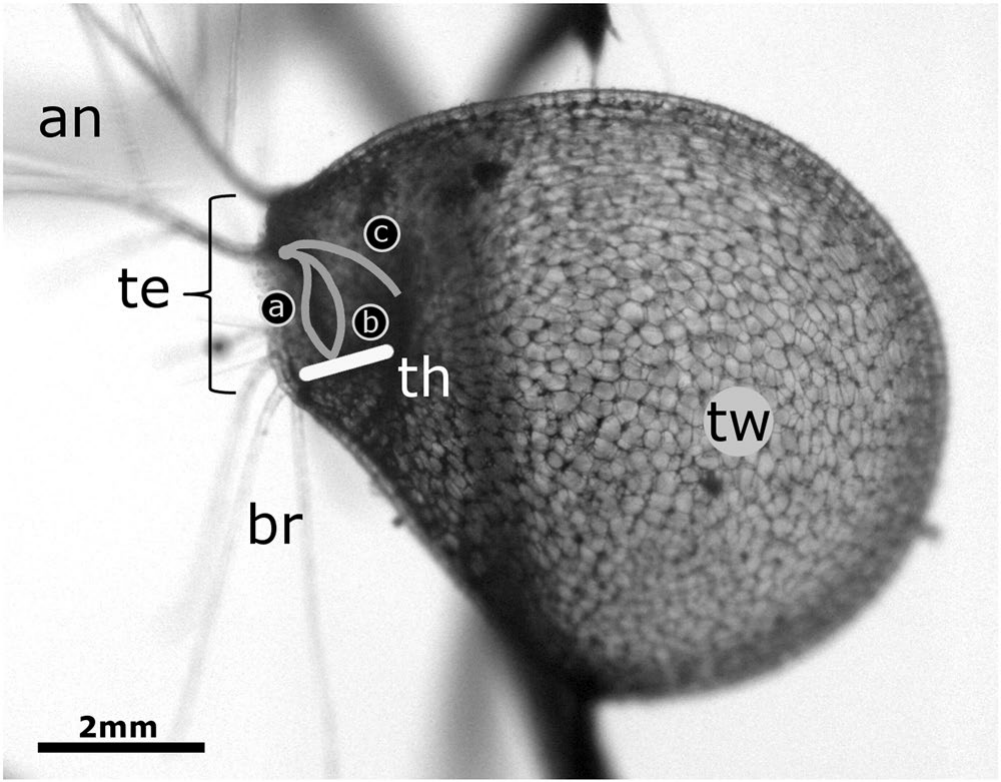
Lateral view on an *Utricularia australis* trap. The trap entrance (te) faces left, and the trigger hairs which protrude from the trapdoor are slightly visible (see Fig. 4 for a SEM image). The trap possesses antennae (an) and bristles (br) for guiding prey organisms grazing algae on the trap towards the entrance, and lateral flexible trap walls (tw). The trapdoor is fixed along the upper part of the trap entrance (its median axes in different phases (**a**–**c**) are indicated as solid grey lines), whereas the motile lower trapdoor edge rests on the threshold (th, indicated by a solid white line). When the trap is set and ready to ‘fire’, the door is (**a**) bulged outwards (convex) and highly sensitive to mechanical perturbations. When prey triggers the trap by touching the trigger hairs, the trapdoor (**b**) becomes ‘unlocked’ by inverting its curvature to concave. Afterwards (**c**), it swings open and water and prey are sucked into the trap. After attaining a phase of maximum opening, the trapdoor re-closes by an inverted motion sequence and finally regains the initial convex curvature.

Aquatic carnivorous bladderworts (*Utricularia* spp., Lentibulariaceae, Lamiales) possess submerged suction traps (‘bladders’) which are the fastest motile trapping devices in the plant kingdom^1, 2^. They are several millimeters long^3^, hollow and water-filled and possess glands which continuously pump water out of the trap lumen by an energy-demanding process^4^. Thereby, a negative hydrostatic pressure is generated inside the bladders and the lateral, flexible trap walls deform and store elastic energy^5–7^. The trap entrance is closed watertight by a trapdoor which possesses several trigger hairs on its outer surface and which is fixed along the upper part of the entrance. The trapdoor rests with its lower free edge on a threshold and is bulged outwards (convex curvature). When prey, predominantly small crustaceans^8, 9^, touches at least one of the trigger hairs, the door rapidly inverts its curvature to concave within ~2 ms (Fig. 1). In this ‘unlocked’ state it cannot resist the water pressure any longer, swings open within ~0.5 ms, the trap walls relax and water and prey is sucked into the bladder within ~1 ms owing to the sudden increase of its volume (the trap ‘fires’) (durations measured for aquatic *U. inflata*)^7^. Suction also occurs spontaneously when the trap is deflated to such an extent that it attains a critical negative pressure inside, where very small perturbations (e.g., mechanical noise) are sufficient to trigger firing^4, 10–12^.

By using tracer particles, Vincent and colleagues (ref. 7) were able to measure a fluid acceleration of 600 *g* during suction until the tracers reached the trap entrance. It was shown by the same authors (with a single recording of a prey capture event) that sucked prey loops inside the trap body. Such a swirling is hypothesized to be crucial for prey retention because traps can capture multiple prey animals successively. In addition to this, the trapdoor re-closes within ~2.5 ms already during the suction process, which is also speculated to be important for avoiding escape of prey and/or an outflow of water enriched with nutrients^2, 7, 13^. Prey dies due to anoxia inside the trap and becomes digested^14^.

Knowledge is generally very scarce regarding how prey organisms of carnivorous plants behave when situated close to the trap or when situated on or inside the trap, how prey organisms trigger the respective capture mechanism (in motile traps), and how the movement of such a trap and of the prey might be interrelated and probably affect each other during capture. For *Utricularia*, little is also known on the effectiveness of the traps and their possible limits regarding capture of relatively large prey^2, 15, 16^. In a broader perspective, such knowledge would indeed be essential to understand trophic interactions (food webs) and possibly to draw ecological and evolutionary conclusions, e.g. regarding cost-benefit ratios and trap and prey (co-)evolution. We investigated the ultrafast trapping mechanism of the Southern bladderwort (*U. australis* R.Br.) in comparison to behavior and movement of one of its natural crustacean prey species, *Ceriodaphnia dubia* Richard (Daphniidae, Branchipoda).

## Results

**Figure 2:**
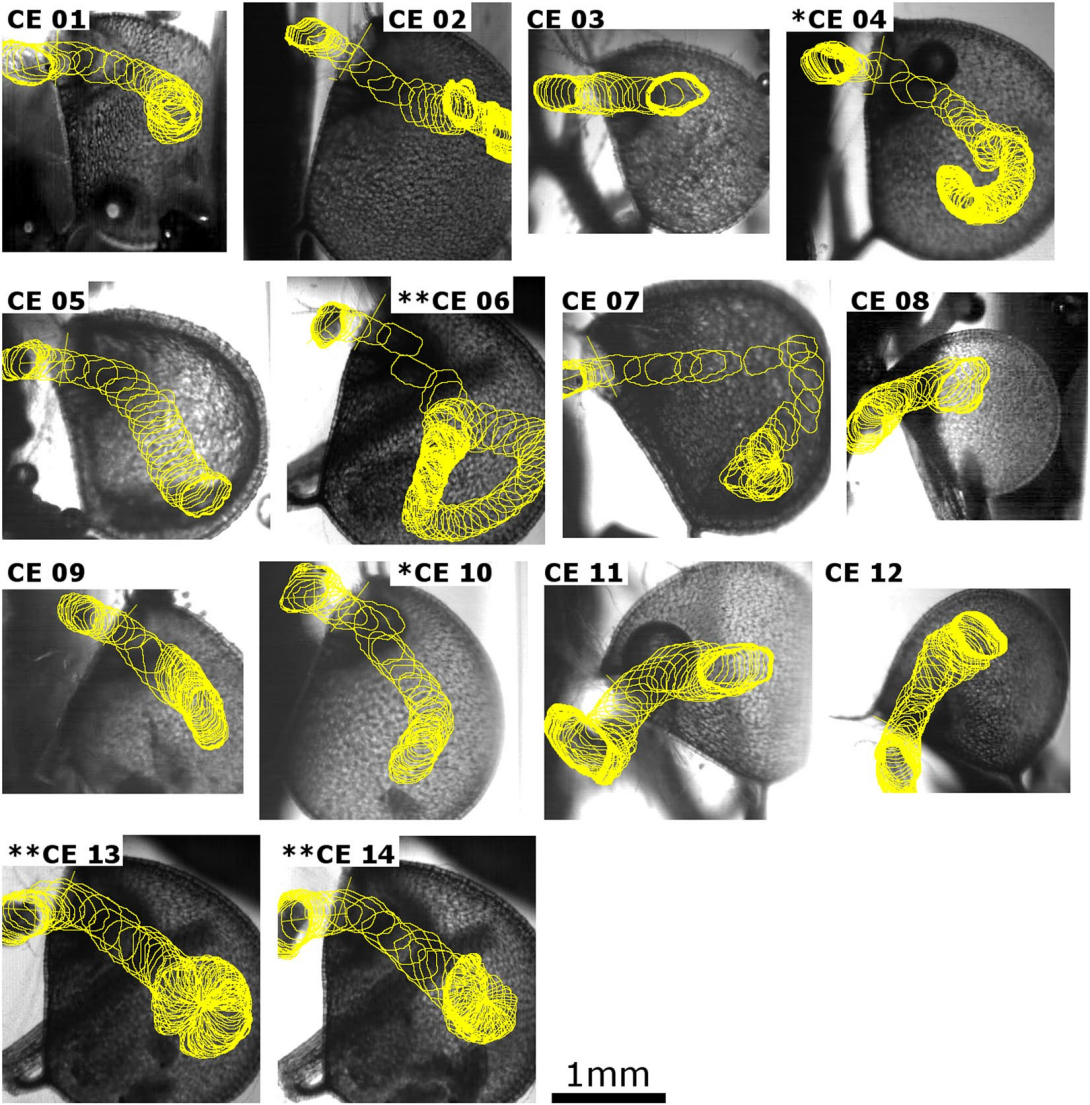
High-speed analyses of *Ceriodaphnia dubia* capture events (CEs) by *Utricularia australis*. The trap entrances face left, and the contours of the prey animals during the CEs are retraced (the time intervals between two frames is 0.1 ms). The prey animals in CEs 06, 07, 13 & 14 loop within the respective traps. CEs marked by a single asterisk (*CEs 04, 10) or with a double asterisk (**CEs 06, 13, 14) were recorded in the same traps, respectively. Brightness and contrast were adjusted for image clarity.

It took between one and 90 minutes after deposition of prey animals into the test chambers until suction occurred, and 14 capture events were recorded (CEs 1–14). In each CE (Movies S1–S14) (Fig. 2), touching the trigger hairs entailed the snap-buckling of the trapdoor, then its inwards swinging, the phase of maximum door opening, and re-closure (outwards swinging), altogether leading to the inevitable and very fast capture of the prey animal. The animals remained motionless inside the trap after capture. After freeing one animal by cutting the respective trap open, it began to swim around, whereas other animals inside the closed traps did no longer move, or performed only weak twitching motions for short periods.

Results (n = 14) regarding prey dimension, trap entrance heights, and prey positions as well as observations on behavior during triggering and suction are listed in Table 1. The mean diameter of the caught prey animal was 0.29 ± 0.04 mm (median: 0.29 mm; IQR: 0.045 mm; min: 0.22 mm; max: 0.39 mm), and their mean length was 0.49 ± 0.08 mm (median: 0.47 mm; IQR: 0.1225 mm; min: 0.34 mm; max: 0.67 mm). The mean height of the trap entrances was 0.51 ± 0.1 mm (median: 0.53 mm; IQR: 0.0875 mm; min: 33 mm; max: 0.72 mm), and the mean ratio between prey diameters and trap entrance heights was 0.6 ± 0.17 (median: 0.62; IQR: 0.1975; min: 0.33, max: 0.94).

**Table 1:** Prey dimensions, trap entrance heights, and prey positions as well as prey behavior during triggering and suction.

Prey capture event	Prey diameter [mm]	Prey length [mm]	Height of trap entrance [mm]	Ratio of prey diameter/trap entrance height	Prey orientation during trap triggering	Structures from prey touching trigger hairs	Prey orientation during suction	Prey action during triggering
CE 01	0.29	0.46	0.53	0.55	Head points towards trap entrance	Head	Head first	Antennae return stroke
CE 02	0.22	0.41	0.55	0.40	Carapace points towards trap entrance	Antennae	Carapace first	Antennae downstroke
CE 03	0.29	0.46	0.37	0.78	Lateral orientation	Antennae	Head first	Antennae return stroke
*CE 04	0.24	0.44	0.51	0.47	Head points towards trap entrance	Antennae	Head first	Antennae downstroke
CE 05	0.28	0.45	0.46	0.61	Head points towards trap entrance	Head	Head first	Forward motion
**CE 06	0.24	0.34	0.72	0.33	Lateral orientation	Antennae	Head first	Antennae downstroke
CE 07	0.26	0.44	0.59	0.44	Head points towards trap entrance	Head	Head first	Forward motion
CE 08	0.31	0.52	0.33	0.94	Lateral orientation	Head	Head first	Forward motion
CE 09	0.31	0.55	0.46	0.67	Lateral orientation	Carapace	Head first	Antennae return stroke
*CE 10	0.28	0.57	0.55	0.51	Lateral orientation	Antennae	Head first	Antennae return stroke
CE 11	0.36	0.67	0.53	0.68	Lateral orientation	Antennae	Head first	Forward motion
CE 12	0.31	0.47	0.4	0.78	Lateral orientation	Antennae	Head first	Antennae return stroke
**CE 13	0.34	0.57	0.54	0.63	Lateral orientation	Head	Head first	Forward motion
**CE 14	0.36	0.57	0.53	0.67	Lateral orientation	Head	Head first	Forward motion

CEs marked by a single asterisk (*CEs 04 & 10) or with a double asterisk (**CEs 06, 13, 14) occurred in the same traps, respectively.

Nine animals were in lateral positions in respect to the trap entrances during triggering events, in four animals the heads and in one animal the carapace were/was orientated towards the trap entrances. They either performed forward motions (six animals), antennae downstrokes (three animals) or antennae return strokes (five animals) during triggering, and touched the bladderwort trigger hairs either with their antennae (seven), heads (six), or with the carapace (one). The one animal whose carapace was orientated towards the trap entrance during triggering became sucked in with the carapace first, all the others with their heads first so that those who possessed a lateral position rotated during the aspiration process (Fig. 2).

In all CEs with ratios of ‘prey diameter/trap entrance height’ ≤0.63 (CEs 01, 02, 04–07, 10, 13), suction of prey was ‘smooth’ and without any visible temporary blockages of the trap entrances or friction-induced slowing-down of prey (Movies S1, S2, S4–S7, S10, S13). In all CEs with a ratio ≥0.67 (max. measured value: 0.94) (CEs 03, 08, 09, 11, 12, 14), friction effects or even temporary trap entrance blockages by prey were visible (Movies S3, S8, S9, S11, S12, S14). Four animals (CEs 04, 06, 13, 14) showed a distinct looping behavior inside the traps (Movies S1, S6, S13, S14), whereas the path of the other animals during suction can be described as being more or less straight to curved (Fig. 2). In all cases, the prey was unable to escape from the traps.

General results of the 14 CEs analyzed regarding prey movement during suction, durations of trap movement phases, and lateral displacement of trap during suction are listed in Table 2. The mean distance prey travelled during suction was 2.4 ± 1 mm (median: 2.2 mm; IQR: 1.1 mm; min: 1.1 mm; max: 4.6 mm). Prey was sucked in at a mean maximum velocity of 2.2 ± 0.8 m/s (median: 2 m/s; (IQR: 0.5 m/s; min: 1 m/s; max: 4 m/s), with phases of very high maximum acceleration (mean: 1100 ± 660 *g*; median: 1150 *g*, IQR: 750 *g*; min: 400 *g*; max: 2800 *g*) which were immediately followed by phases of very high maximum deceleration inside the bladders (mean: (−940 ± 520 *g*); median: (−800 *g*), IQR: (−700 *g*); min: (−400 *g*); max: (−1900 *g*)). The mean duration of trapdoor snap-buckling after triggering was 1.5 ± 1 ms (median: 1.3 ms; IQR: 1.275 ms; min: 0.4 ms; max: 3.7 ms), that of trapdoor inward swinging until maximum opening was 0.9 ± 0.4 ms (median: 0.9 ms; IQR: 0.3 ms; min: 0.3 ms; max: 1.7 ms), that of maximum door opening was 1.7 ± 1 ms (median:1.2 ms; IQR: 0.775 ms; min: 0.9 ms; max: 4.1 ms), and the duration of trapdoor re-closure was 6.5 ± 3.8 ms (median: 4.5 ms; IQR: 6.45 ms; min: 1.6 ms; max: 12.9 ms). The mean duration of the suction process (i.e., the duration of prey capture, which is the time from trapdoor opening until full re-closure) was 9 ± 3.3 ms (median: 8.3 ms; IQR: 5.775 ms; min: 5.2 ms; max: 14.9 ms). During suction, traps performed a ‘forward’ motion in direction to the prey and thereby became laterally displaced by 0.11 ± 0.06 mm in mean (median: 0.09 mm; IQR: 0.0725 mm; min: 0.05 mm; max: 0.26 mm) (n = 12, only measured for CEs 01–12, see Table 2) (e.g., Movie S7). After reaching the maximum displacements, the traps swung back to their initial positions.

**Table 2:** Prey movement, durations of trap movement phases, and lateral displacements of traps.

Prey capture event	Prey movement			Trap movement					
	Distance travelled by prey during suction [mm]	Max. velocity of prey [m/s]	Max. acceleration/deceleration of prey [*g*]	Duration of trapdoor snap-buckling [ms]	Duration of trapdoor inward swinging until maximum opening [ms]	Duration of maximum trapdoor opening [ms]	Duration of trapdoor re-closure [ms]	Duration of the suction process [ms]	Lateral displacement of trap during suction [mm]
			Duration from triggering until reaching *g*_max_/*g*_min_ [ms]						
**CE 01**	2.0	2	800/−600	1.8	0.9	1.1	3.8	5.6	0.10
			2.95/3.15						
**CE 02**	2.3	2.5	1300/−1200	0.4	1.2	1.2	8.4	10.8	0.14
			1.65/2.05						
**CE 03**	1.4	2	1500/−800	0.4	0.8	1.1	3.7	5.6	0.10
			2.05/2.35						
*CE 04	3.5	3	1800/−1600	3.2	0.9	0.9	5.1	6.9	0.15
			4.55/4.75						
**CE 05**	2.2	2.5	1100/−800	1.5	0.5	1	9.7	11.2	0.07
			2.55/2.65						
**CE 06	4.6	3	1200/−1800	0.9	0.6	1.2	10.1	11.9	0.05
			1.85/2.15						
**CE 07**	3.9	4	2800/−1900	1.0	0.6	1.1	11.1	12.8	0.26
			2.15/2.35						
**CE 08**	1.1	1	700/−500	1.1	0.6	3	1.6	5.2	0.07
			4.05/4.35						
**CE 09**	1.6	2	1200/−500	0.4	1.4	2	3	6.4	0.06
			2.35/2.55						
*CE 10	2.2	2.5	1300/−1200	3.7	0.9	1.4	3.5	5.8	0.16
			5.75/6.05						
**CE 11**	1.8	1.5	400/−900	2.0	0.3	3	3.5	6.8	0.08
			4.55/4.75						
**CE 12**	1.6	1	500/−400	0.6	1.7	4.1	3.8	9.6	0.08
			5.85/6.05						
**CE 13	2.8	2	400/−500	2.0	0.8	1.5	10.5	12.8	—
			3.45/3.85						
**CE 14	2.6	2	400/−400	1.5	0.9	1.1	12.9	14.9	—
			3.25/3.55						

CEs marked by a single asterisk (*CEs 04, 10) or with a double asterisk (**CEs 06, 13, 14) occurred in the same traps, respectively. See Fig. 1 for schematic representation of the trapdoor movement phases.

The trap where the highest values for prey acceleration during suction (~2800 *g*), for subsequent deceleration inside the bladder of about (−1900 *g*), and for prey velocity (~4 m/s) were measured (CE 07, Movie S7), was to a great extent filled with air in the deflated (ready-to-catch) state. During suction, a water jet can be seen travelling in a straight manner from the trap entrance region to the rear trap wall, where it splashes. Such a jet is also visible in CE 05 (Movie S5). All other traps were (mainly) water-filled in the deflated state, and water jets could hence not be observed during suction. In this group of traps, maximum prey velocities of ~3 m/s during suction and maximum accelerations of ~1800 *g*/~1200 *g* and decelerations of ~(−1600 *g*)/~(−1800 *g*) (CEs 04 & 06) were measured. In CEs 04, 10 & 11, small air bubbles were visible in the upper entrance regions inside the bladders, which noticeable interfered with the trapdoors during their opening sequences (Movies S4, S10 and S11). More precisely, the bubbles appeared as stuck in the entrance regions and they became pushed towards the trap lumina during the inward motions of the doors. In CEs 04 & 10, the bubbles detached from the entrance region during prey capture and freely floated inside the bladders afterwards. The bubble in CE 11 continued to adhere to the entrance during and after prey capture and became strongly deformed by the inflow of water and by the impact of the sucked animal (Movie S11).

**Figure 3:**
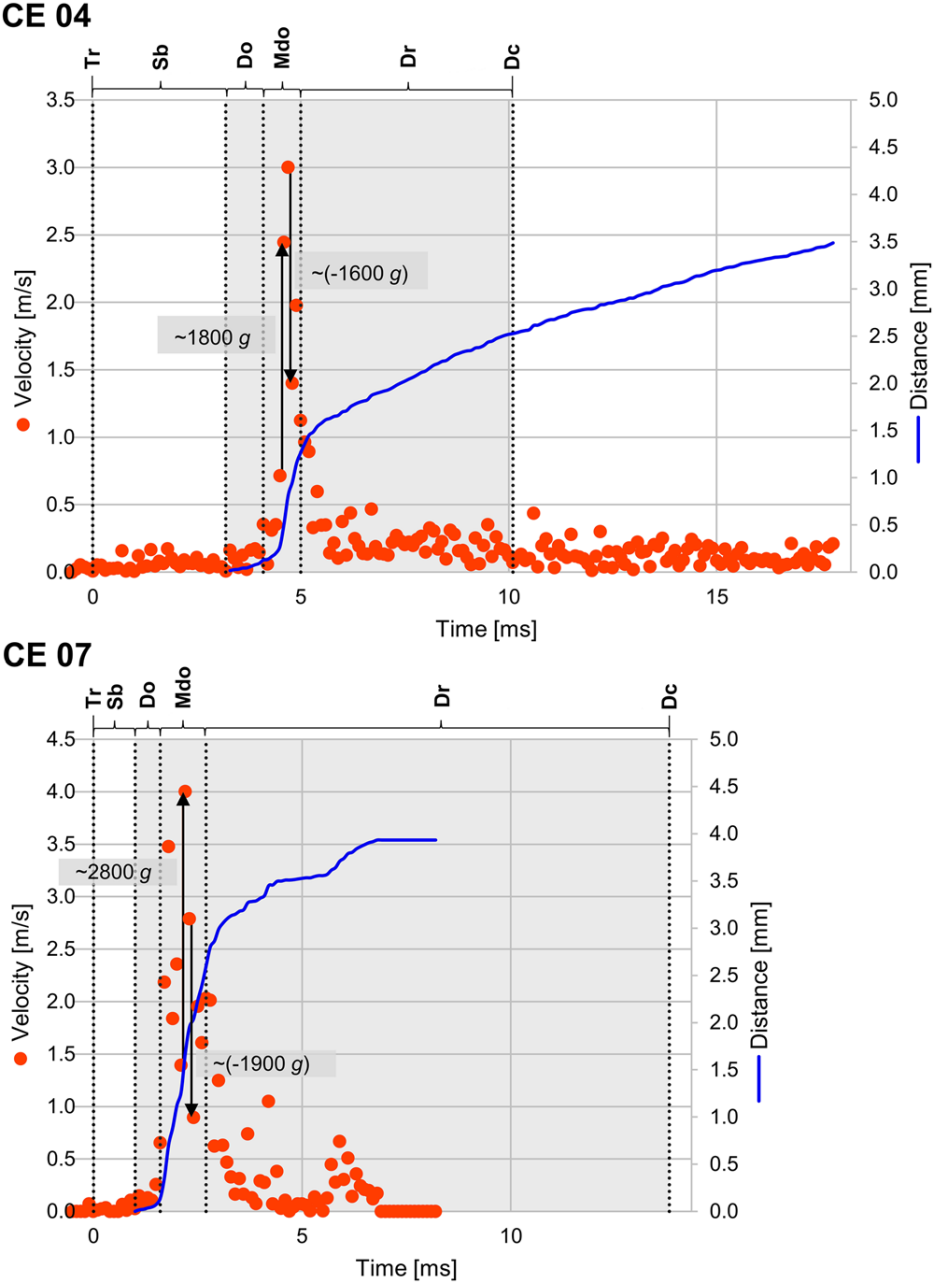
Interrelations of prey and trap movement steps and phases, exemplarily depicted for CE 04 and CE 07. Velocity of captured prey animals (red dots) and distance travelled by captured prey animals (solid blue line) are depicted over time, with maximum acceleration *g*_max_ and maximal deceleration *g*_min_ indicated by black arrows. On the upper margins of the graphs, the trap movement steps/phases are indicated: trap triggering (Tr), snap-buckling of the trapdoor (Sb), door opening (Do), phase of maximum door opening (Mdo), door re-closure (Dr), and the point in time when door is fully closed again (Dc). The phases in which suction took place are grayed out. The interrelations for the other CEs can be seen in Figures S1–S12.

The interrelations of prey and trap movement steps and phases are depicted in Figs 3 and S1–S12. The maximum values for prey acceleration and deceleration were all reached in the phases of maximum door opening, except for CEs 03, 10 & 15 where the maximum deceleration values were measured at the beginnings of the respective door re-closure phases. The maximum prey accelerations occurred during the onset of suction, mainly when the prey passed the narrow trap entrance region, whereas the maximum decelerations occurred inside the trap bodies. During onset of suction, also most of the distances the prey animals passively travelled during the suction process were covered, with the most notable exceptions of CEs 04 & 06 where the animals travelled notably further during the door re-closure phases (Figs 3 and S5). In CE 04, the prey even continues to travel after full door re-closure.

The trigger hairs of the *Utricularia* bladders may undergo strong bending deformation during the capture motion. An unfolding process of one trigger hair from a kinked state back to a straight state during door-re-closure can be seen exemplarily in Movie S15. This behavior was observed in three CEs in total (CEs 02, 07, 10).

## Discussion

Until now, there are only few reports available which (superficially) deal with the behavior of prey in the proximity of a motile or non-motile carnivorous plant’s trap and during trap triggering (in motile traps) and tackle the question how the trap structures interact with the animal during capture^17–19^. For the very most part, analyses of trap kinematics were performed with artificially triggered traps^7, 20–22^. Although we observed prey capture events also under laboratory conditions, our attempts are the first which incorporate behavior and movement of the prey and the motion of the trap. Such observations of very fast organisms and structures would be very difficult to perform in the field, regarding e.g. the aquatic nature of the organisms investigated and their small sizes, the required video frame capture speed, frame exposure time, and illumination.

We have biomechanically analyzed 14 capture events of *C. dubia* by *U. australis*. The suction dynamics of the *Utricularia* trap depends primarily on the underpressure generated inside the bladder (apart from morphological factors like the entrance width), a dependency which appears evident but which has not yet been investigated experimentally. Due to methodical difficulties, it was neither possible for us to determine the underpressure in the traps tested here, nor to evaluate their exact three-dimensional shapes in detail. Hence, the gained biomechanical data represent snapshot values for rather undescribed conditions of trap deflation, which might (partly) explain the differences among the data (e.g., regarding the different values measured for the otherwise identical traps in some of our experiments). Future attempts could either try to record the underpressure values inside the bladders simultaneously to the prey capture experiments (cf. refs 5, 6, 12 and 23), or attempt to evaluate suction dynamics by analyzing spontaneous firings, i.e. firings at trap states of critical underpressure without prey touching the trigger hairs. Spontaneous firings occur naturally^4, 10, 11^ but can also be artificially initiated by evacuation of water from the trap lumen with fine capillaries^12^. By this, a fairly reliable and recoverable experimental trap status could be installed.

By assessing prey capture rates with natural and manipulated traps of aquatic *Utricularia vulgaris*, Meyers and Strickler^16^ found that substrate-dwelling copepod prey (*Chydorus sphaericus*) is guided by the bristles and antennae (see Fig. 1) towards the trapdoor and captured. Also, Harms and Johansson^8^ found a ‘preference’ for substrate-dwelling cyclopoid species as prey in *U. vulgaris*. Such a guiding can most likely be excluded for *C. dubia* tested here, as this species is not a substrate-dweller but rather a planktonic filter feeder^24, 25^. However, *Ceriodaphnia* is reported as bladderwort prey in the literature^9, 26, 27^, which is in general agreement with our own snapshot prey analysis (see Materials & Methods and Table S1). The trigger hairs on the trapdoor protrude into the water in front of the trap entrance so that passing animals can easily touch these structures and trigger suction. In our analysis, no *C. dubia* individual was able to escape the suction stream once the respective trap was triggered. Hence, the orientation and action of the animal during triggering had no influence on the accomplishment of successful capture. The looping behavior inside the bladders, which is hypothesized to be crucial to avoid prey escape^7^, was observed in four CEs and had no effect on prey capture and retention in direct comparison to the other 10 CEs where looping did not occur. Therefore, looping is presumably not an evolutionary advantageous ‘feature’ of the trap improving prey retention but rather a side effect depending on suction dynamics, trap and prey size and shape, as well as other factors.

In our experiments, small prey became sucked in very smoothly, but also bigger prey animals with ‘prey diameter/trap entrance height’ ratios of up to 0.94 were successfully captured. In the latter cases, visible friction effects and also temporary blockages of the entrance regions by the prey did not prevent their capture, which indicates that the suction force was high enough in each case to cope with the opposing forces. Indeed, in CE 08 where such temporary blockage can be noted, the duration of suction (i.e., the trapping duration) is even shortest (5.2 ms) among all CEs. It remains to be investigated if also a potential deformation of the *C. dubia* carapace during passage through the trap entrance region plays a role (see Kruppert and colleagues^28^ for a biomechanical analysis of the *Daphnia pulex* carapace). It is supposable (but not observed in this study) that *C. dubia* individuals exist which are too big to get sucked into the trap, and that traps at early stages of deflation (with low underpressure values inside) cannot cope with prey blocking the entrances.

The interrelations of prey and trap movement steps and phases of all recorded CEs are homogeneous, without any great deviation from a general sequence (Figs 3 and S1–S12). The onset of suction, with the phases of (maximum) trapdoor opening, is characterized by a strong acceleration of the prey, followed by a strong deceleration inside the bladder and the irrevocable capture after trapdoor re-closure. Prey travels ~2.4 mm during capture, and developing swirls and streams inside the trap are likely to carry the animal further even after termination of the suction process, i.e. after trap door closure. Suction lasts ~9 ms, corroborating the description of *Utricularia* as being the fastest motile carnivorous plant in terms of capture speed^7^, followed by the Waterwheel plant (*Aldrovanda vesiculosa*, Droseraceae) with underwater snap-traps which snap within 20 ms^29^. It is interesting to note that two of the fastest motions to be found in the plant kingdom^30^ are performed by the traps of aquatic carnivores.

Vincent and colleagues^7^ measured a maximum fluid velocity of ~1.5 m/s and acceleration of ~600 *g* during suction in *U. inflata*, but the tracer particles used could only be followed until they reached the trap entrance region where they were then obscured. Although our measurements are not directly comparable (we tracked prey animals), we show that, in principal, (much) higher values for velocity (max. 3–4 m/s) and acceleration (max. 1800–2800 *g*), depending on the status of the trap (e.g., water- or air-filled), can be achieved. Due to the small diameter of the trap entrance region and the concomitant increase of flow velocity, it is not surprising that the maximum prey acceleration was measured in this region. We assume that the abrupt deceleration (max. (−1800 *g* to −1900 *g*)) is due to the fact that the rapidly travelling prey and the accompanying water ‘collide’ with a stationary or, at least, slower fluid body inside the bladder. Thereby, the entire kinetic energy is dissipated through viscous effects when the fluids mix^31^, considering that the fluid within the bladder often contains (partly) digested prey which probably adds to its viscosity. Because of the fact that the animals were motionless after capture and appeared as dead or stunned, it is supposable that the sequence of acceleration and deceleration leads to mechanically evoked immobilization of prey. If not dead yet, anoxia^14^ finally leads to the death of the immobile small crustaceans in the closed trap. Other animals are reportedly still agile after capture^15^, so that it is up to future investigations to evaluate if the forces acting on the animals during suction lead to lethal internal and/or external structural damage.

We observed that air bubbles mechanically interfere with moving trap structures and prey during the capture process. It can be assumed that these bubbles stick to small structures, e.g. glands, on the entrance region and non-critically (in terms of successful capture) slow-down the motion of the trapdoor and/or of prey. In CEs 04, 10 and 11 (Movies SS4, S10 and S11), it can be speculated that the process of the door curvature inversion is slowed-down by such bubbles. On the other hand, we also find comparably slow curvature inversion processes in other CEs (Movies S1, S13, S14) where no such bubbles can be seen. Probably, in these traps the underpressure inside was relatively low, leading to a slower door curvature inversion.

Traps which fire in air are described to be short-circuited if they contain too large air bubbles afterwards^2, 32^. We find that almost completely air-filled bladders of *U. australis* (in CEs 05 & 07, Movies S5 and S7) are apparently able to reset to a deflated state and to fire. This shows that the bladders are much more functionally resilient to mechanical stresses and (a)biotic perturbations they experience in their habitat (changing water availability, water streams, contact to large animals etc.) than previously thought. If this finding represents a permanent and genus-wide feature, if it depends on the vigor of the trap and/or of the whole plant, and how this process is physiologically achieved are matters for future investigations. Probably, active underpressure adjustments inside the bladder and dissolution of a proportion of the air in the remaining water (which then becomes pumped out) occurs. However, the observed fluid jet streams in the above mentioned (partly) air-filled traps travel through air, with no stationary water bodies acting as ‘barriers’ inside the bladders. The (nearly) straight trajectories of the jets indicate that the swirls, which may develop inside ‘typical’ fluid-filled bladders (CEs 04, 06, 13, 14), are probably evoked by the interplay of the sucked-in water and the fluid body inside the trap.

**Figure 4:**
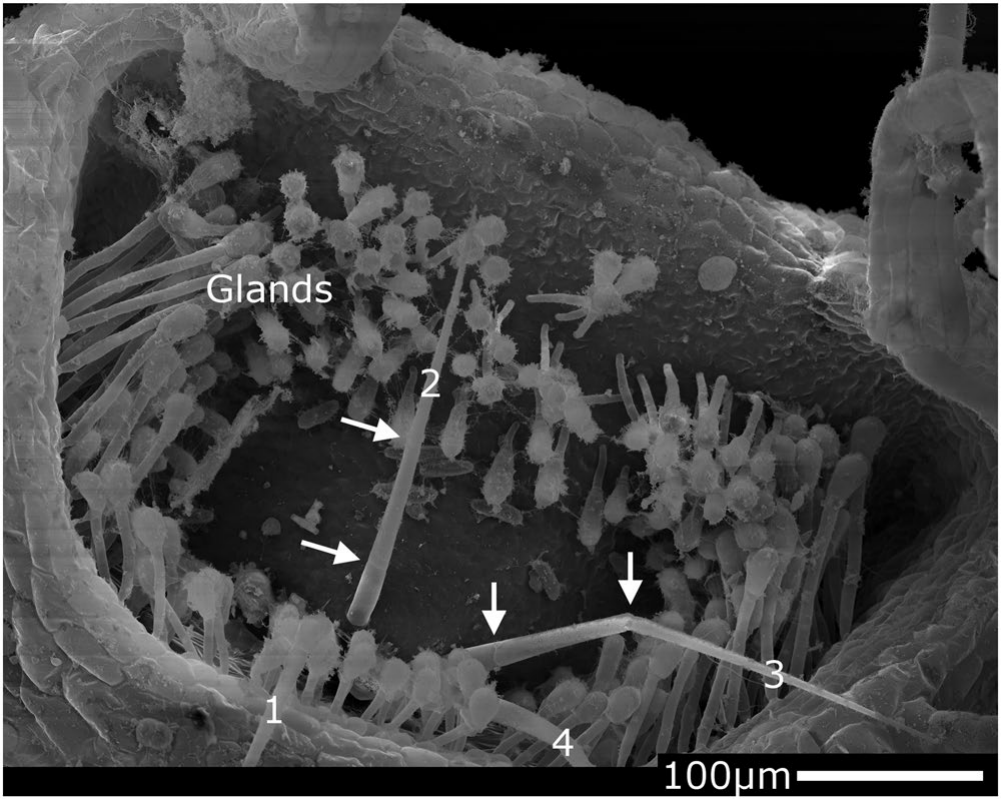
Trigger hair bending deformation. SEM micrograph of the outer trapdoor surface. A multitude of glands of uncertain function (cf. ref. 2) as well as the four trigger hairs (1–4) – which protrude from the door – are well visible. The arrows in trigger hairs 2 and 3 indicate cell-cell-junctions, which we hypothesize to act as hinge zones.

The sequence of motions of the trap and trapdoor during trap firing and the durations of the individual processes are similar as reported by Vincent and colleagues^7^. We additionally observed a ~0.1 mm ‘forward’ motion of the entire trap during suction, which is evoked by the trap movement in interplay with the inertia of the water (the trap displacement is in opposite direction to the stream of the sucked water) and further enabled by the flexible connection of the trap to the plant. Individual trap characteristics (underpressure value, counter-acting trap mass which also depends on the fact if the trap is air- or water-filled) are likely to dictate the extent of motion. Although this displacement represents only a small fraction of the ~2.4 mm the prey travels during capture, the distance of the prey to the trap entrance at the time of triggering is given by the only a few 100 µm long trigger hairs (cf. Fig. 4 and ref. 2). Hence, the ‘forward strike’ of the trap can indeed be assumed to help in overcoming a putatively critical flight distance of the prey.

We also observed a striking deformation of trigger hairs during door movement. According to Vincent and colleagues^7^ the hairs flap against the trapdoor in a specific manner to not block the trap entrance during suction. This flapping is evoked and dictated by the kinematics of the door, and no deformation of the hairs themselves has yet been described. The here observed bending is most likely due to the water flow and/or caused by collision with the sucked-in prey. Probably, the deformability is a safety feature to prevent fracture during suction, which has similarly been hypothesized to be present in the deformable trigger hairs in snap-traps of the aquatic Waterwheel plant (*Aldrovanda vesiculosa*)^33^. *Utricularia* trigger hairs consist of several elongated cells^13^, and we suspect that the junction zones between the cells may act as hinge zones (Fig. 4).

The aquatic bladderwort suction trap is a functionally resilient structure for reliably capturing zooplankton prey. It can be assumed that the only countermeasures of *C. dubia* to avoid capture by *U. australis* suction traps are encounter avoidance, a structural barrier making them unfit to pass the trap entrance, and/or an effective flight response. Encounter avoidance could, for example, be realized by an altered behavior in terms of swimming speed and/or an altered aggregation behavior, i.e. swimming in distance to plants. Such reactions are already described in the Daphniidae as inducible defenses against animal predators provoked by chemical cues^34^. Also, inducible defense reactions like an alteration of the shape/dimensions of their bodies could support to impede suction, e.g. by increasing the body dimensions above the trap door diameter. Apparently, the mechanical contact to the trigger hairs and the process of trapdoor snap-buckling (which is accompanied by only small water displacements) do not induce flight responses of the prey. Probably, the timescales of both processes are too short to be processed fast enough by the animal’s nervous system. Also, we did not observe attempts of the animals to swim against the suction streams, which again would presuppose a processing and orientation of the body opposite to the torrent *Utricularia* produces. Evolving a fast enough sensory and reaction system triggered upon certain mechanical stimuli (trigger hair contact, sensing of a water flow field induced by snapping trapdoor) would probably allow for such a flight response. However, *C. dubia* is regarded as a slow swimmer^35^ and, especially, the reaction speed of the arthropod nervous system cannot be reduced unlimitedly (e.g. due to the absence of Schwann cells in arthropods). Careful observations of the crustacean prey and experimental evaluation of the countermeasures are promising subjects for future studies.

## Materials and Methods

### Plant cultivation, prey animal selection and culture

*U. australis* plants were initially purchased from Gartenbau Thomas Carow (Nüdlingen, Germany). The plants used for the prey capture experiments during March-July 2016 were cultivated outdoors in the Botanic Garden Freiburg, Germany. A 40 l plastic container filled with rain water and with dried *Carex* spec. leaves as substrate was used. The test plants grew together with *Salvinia* spec. and were shaded with a plastic net to avoid overheating on hot summer days. The water also contained a multitude of small crustaceans as prey.

During a snapshot prey spectrum analysis of 86 traps (collected 01.10.2015) from an *U. australis* population growing in a pond in the city of Gelsenkirchen, Germany (51°30′17.9″N and 7°04′58.7″E) (Table S1), *C. dubia* was recorded as natural prey. It was chosen as test species for this study because it is a planktonic filter feeder^24, 25^. In contrast to substrate-dwellers, it does not crawl and graze algae on *Utricularia* and is not guided by trap appendages towards the entrances^16^. The pond has a maximum depth of 40 cm and is located on a sunny park site with further ponds containing *U. australis*. In addition to the bladderwort, *Typha* sp. and *Caltha palustris* were growing in the pond. *C. dubia* occurred together with *C. reticulata*, *Chydorus sphaericus*, *Eucyclops serrulatus*, *Eudiaptomus gracilis*, *Herpetocypris reptans*, *Notodromas monacha*, and *Simocephalus vetulus*.

Consequently, *C. dubia* was brought into culture. The daphniids were kept in 1 l beakers (J. WECK GmbH and Co. KG, Wehr, Germany) filled with charcoal filtered tap water in a climate chamber at 20 ± 1 °C and a day-night rhythm of 16:8 h (light:dark) in the Department of Animal Ecology, Evolution and Biodiversity of the Ruhr-University Bochum, Germany. Every two days, *Scenedesmus obliquus* was added *ad libitum* to feed the daphniids. In the same rhythm, algal remnants, exuviae and resting eggs were removed to keep a clonal culture with only asexual parthenogenetic reproduction. Animals of different ages (2–5 days) were sent to the Plant Biomechanics Group (Botanic Garden, University of Freiburg, Germany), there transferred into jars filled with tap water and used for the prey capture experiments.

### Prey capture experiments

Capture experiments were performed at room temperature in the microscopy lab of the Plant Biomechanics Group Freiburg. Leaf fragments with single, empty traps (no prey item visible) were cut-off from the plants and glued to a hollow needle (without syringe) with underwater adhesive (Dupla DekoFix liquid, Dohse Aquaristik GmbH & Co. KG, Grafschaft-Gehrdorf, Germany). Polystyrene cuvettes (4 ml volume, Rotilabo, Carl Roth GmbH + Co. KG, Karlsruhe), which are otherwise commonly used for spectroscopic experiments, were glued onto microscopy slides for stability and filled with the tap water already used in the *C. dubia* culture. The needles with the traps were then carefully placed in the water-filled ‘test chambers’ (the cuvettes), with the syringe connectors facing upwards. *C. dubia* animals were then carefully transferred into the test chambers with a pipette, and the syringes were connected to the hollow needles for adjusting (i.e., lowering) the water-level.

A high-speed camera (Motion Pro Y4, IDT Inc., Tallahassee, FL, USA) in combination with a stereo microscope (SZX7, Olympus Corp., Tokyo, Japan) and a cold light source (techno light 270, Karl Storz GmbH & Co. KG, Tuttlingen, Germany) were utilized to record CEs from lateral views with a recording speed of 10,000 fps. The software Motion Studio (version 2.10.05, IDT Inc.) was used for data acquisition. Two traps captured several prey animals consecutively (indicated by asterisks in figures and tables). A graticule calibrated to 1 mm (Pyser-SGI Ltd., Edenbridge, UK) served as scale for calculating the thicknesses and lengths of the prey animals, the heights of the trap entrances, and, as described in the following, for analyzing the prey and trap movements during suction.

In the prey capture videos gained, the contours of each animal during each time step were retraced (Fig. 2) and the centroids were calculated and tracked in Fiji/ImageJ^36^. The distances travelled (beginning from trapdoor opening) as well as velocities and accelerations of the prey during suction were then calculated. The results of the prey motion sequence analyses were aligned to the following points in time and processes/phases during capture motions of the respective bladderwort traps (see also Fig. 1): (1) Touching of trigger hairs (triggering of trap movement), (2) process of door curvature change from convex to concave, (3) process of trapdoor opening (inward-swinging), (4) phase of maximum trapdoor opening, (5) trapdoor closure (outward-swinging) and changing of door curvature from convex to concave. The trapdoors were visible in each video through the translucent lateral trap entrance walls, and the motions could be tracked. In some cases, brightness and contrast of the movie frames were adjusted for clarity. In addition to these comparative prey-trap movement analyses, the following aspects regarding the prey animals were additionally noted: (1) Position of the animal in respect to the trap entrance prior to triggering, (2) organ/structure of the animal touching the trigger hairs, (3) movement of the animal during triggering, and (4) position of the animal in respect to the trap entrance during suction.

### Trigger hair deformation

Additional observations on bladderwort trigger hair deformation were obtained from the prey capture videos mentioned above. We also performed SEM analyses of trigger hairs with a LEO 435 VP (Leica Corporation, Wiesbaden, Germany). The specimen investigated was methanol fixated^37^, then critical point dried (CPD 030, BAL-TEC Inc., Germany), mounted on an aluminum stub by using conductive double-sided adhesive tabs (Plano GmbH, Wetzlar, Germany), and gold-sputtered (approx. 15 nm) (Sputter Coater 108 auto, Cressington Scientific Instruments Ltd., Watford, UK).

## Electronic supplementary material

Supplementary Information File

Movie S1

Movie S2

Movie S3

Movie S4

Movie S5

Movie S6

Movie S7

Movie S8

Movie S9

Movie S10

Movie S11

Movie S12

Movie S13

Movie S14

Movie S15
