# Supplementary material for: Biomechanical analysis of prey capture in the carnivorous Southern bladderwort (Utricularia australis)
Source: Sci Rep. 2017 May 11;7:1776. doi: 10.1038/s41598-017-01954-3 (PMC5431978; doi:10.1038/s41598-017-01954-3)
Supplement: Supplementary file 1 — Supplementary Information File [file 41598_2017_1954_MOESM1_ESM.pdf]

SUPPLEMENTARY INFORMATION FOR

Biomechanical analysis of prey capture in the carnivorous Southern bladderwort (*Utricularia australis*)

Simon Poppinga[1,*], Lars Erik Daber[1], Anna Sophia Westermeier[1,2], Sebastian Kruppert[3], Martin Horstmann[3], Ralph Tollrian[3], Thomas Speck[1,2]

[1]Plant Biomechanics Group, Botanic Garden, University of Freiburg, Schaenzlestrasse 1, D-79104 Freiburg im Breisgau, Germany.

[2]Freiburg Centre for Interactive Materials and Bioinspired Technologies (FIT), Georges-Koehler-Allee 105, D-79110 Freiburg im Breisgau, Germany.

[3]Department of Animal Ecology, Evolution and Biodiversity, Ruhr-University Bochum, Universitaetsstrasse 150, D-44780 Bochum, Germany.

**Corresponding author: simon.poppinga@biologie.uni-freiburg.de*

Content:

- Figures S1-S12
- Table S1
- Legends for Movies S1-S15

Figures S1-S12:

In the following, the Interrelations of prey and trap movement steps and phases of all recorded CEs except for CE 04 and CE 07 (Figure 3) are presented. Velocity of prey (red dots) and distance travelled by prey (solid blue line) are depicted over time, with maximum acceleration g_{max} and maximal deceleration g_{min} indicated by arrows. On the upper frames, the trap movement steps/phases are indicated: trap triggering (Tr), snap-buckling of the trapdoor (Sb), door opening (Do), phase of maximum door opening (Mdo), door reclosure (Dr), and the point in time when door is fully closed again (Dc). The phases in which suction took place are grayed out.

CE 01

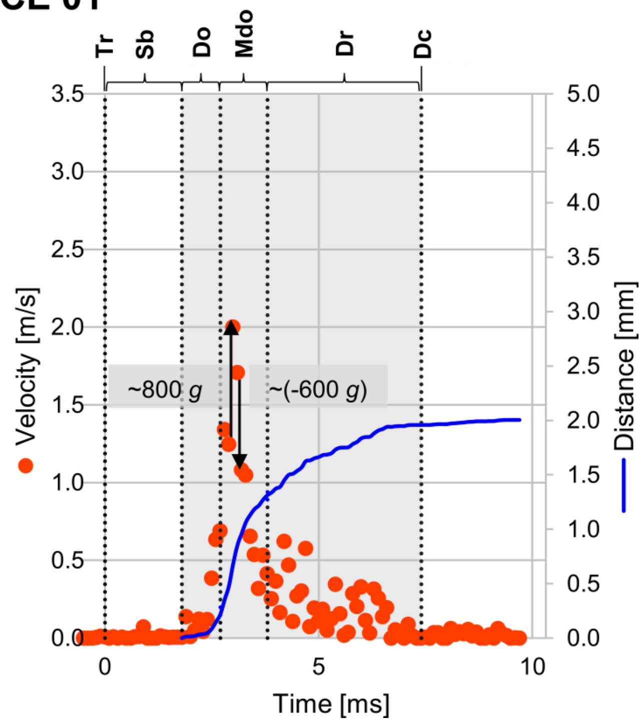

Figure S1: Interrelations of prey and trap movement steps and phases of CE 01.

CE 02

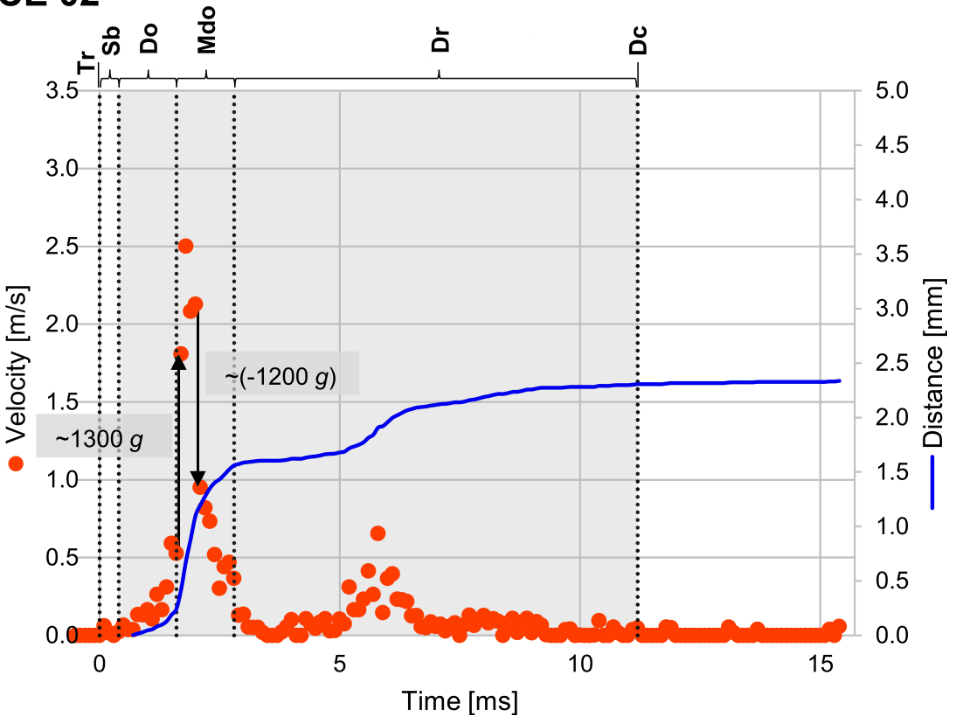

Figure S2: Interrelations of prey and trap movement steps and phases of CE 02.

CE 03

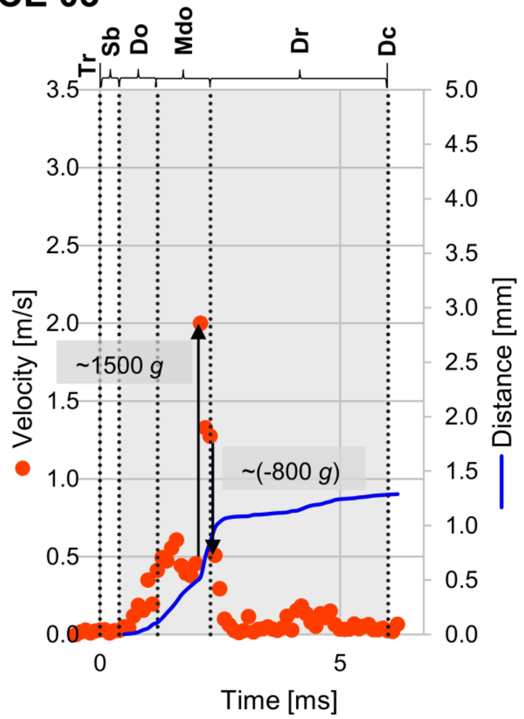

Figure S3: Interrelations of prey and trap movement steps and phases of CE 03.

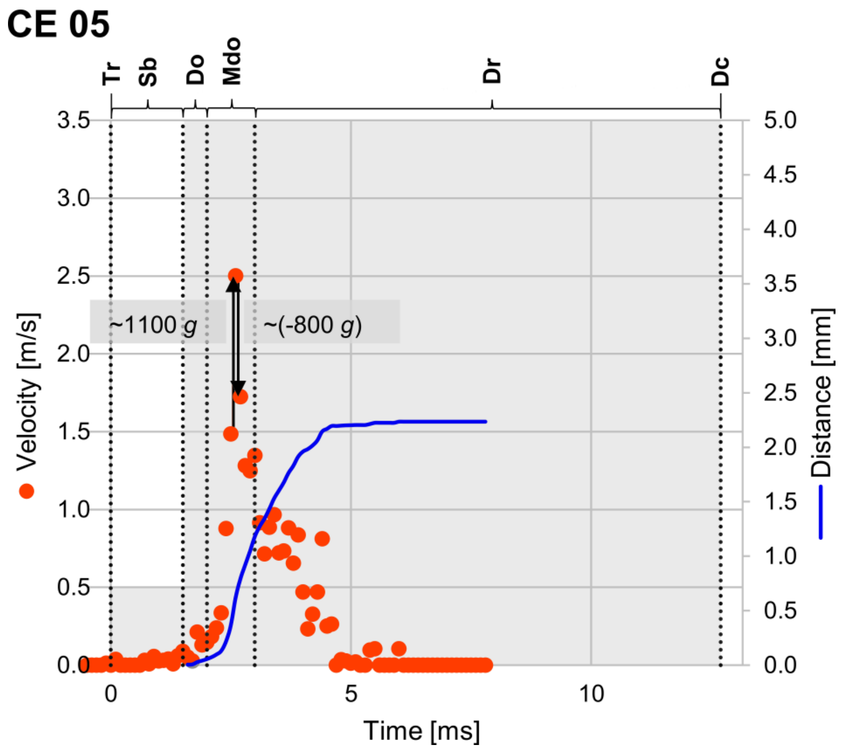

Figure S4: Interrelations of prey and trap movement steps and phases of CE 05.

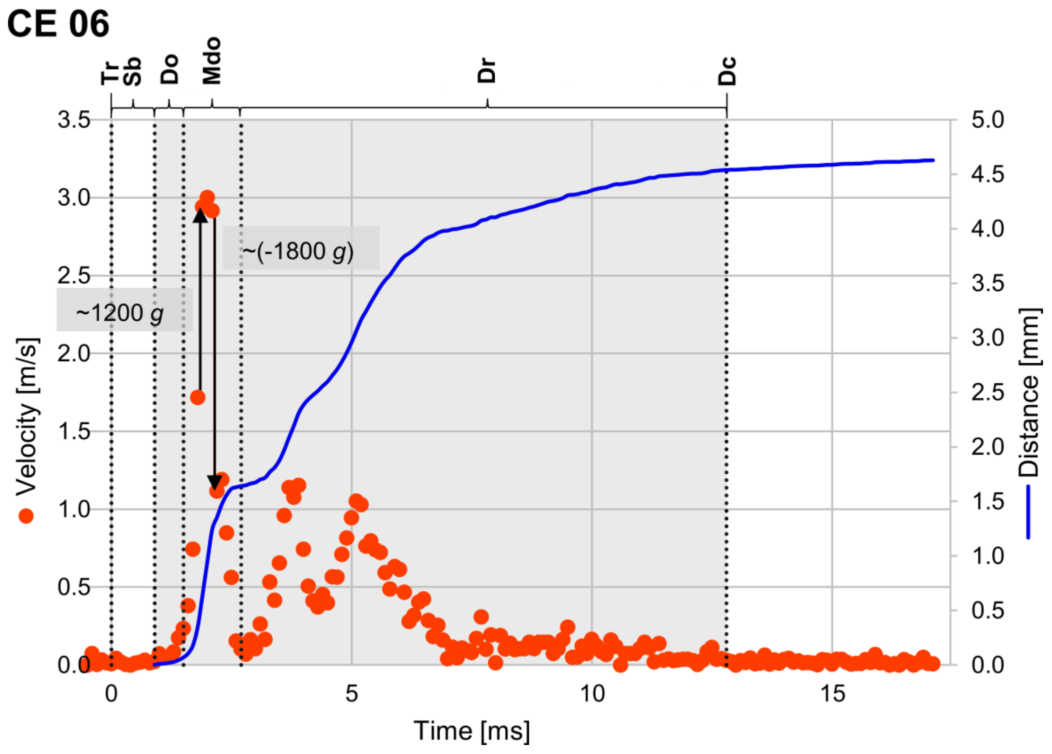

Figure S5: Interrelations of prey and trap movement steps and phases of CE 06.

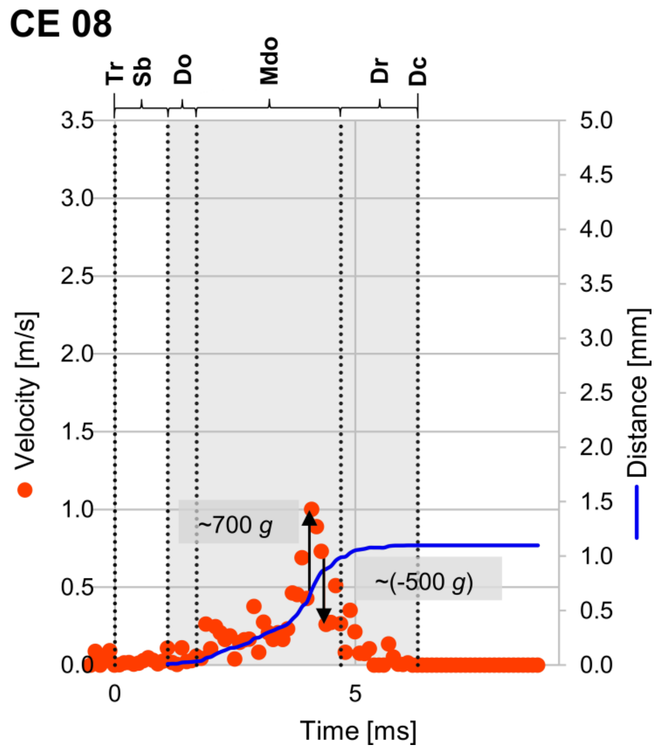

Figure S6: Interrelations of prey and trap movement steps and phases of CE 08.

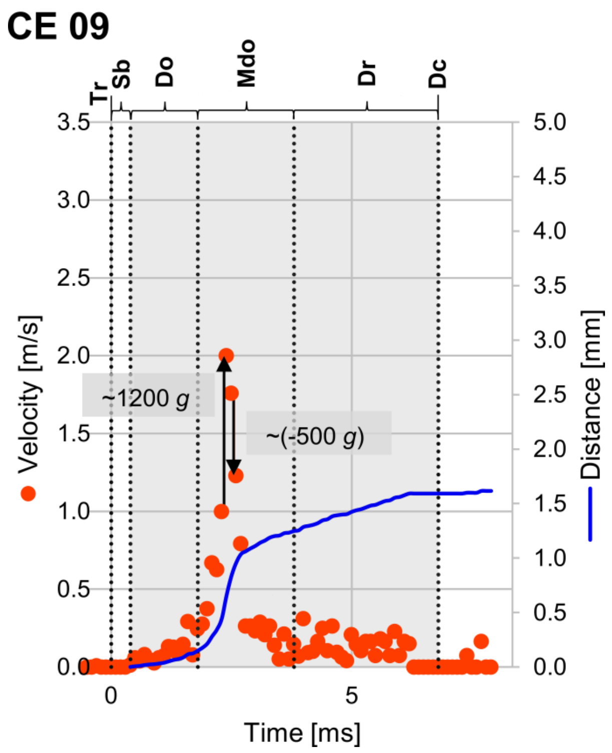

Figure S7: Interrelations of prey and trap movement steps and phases of CE 09.

CE 10

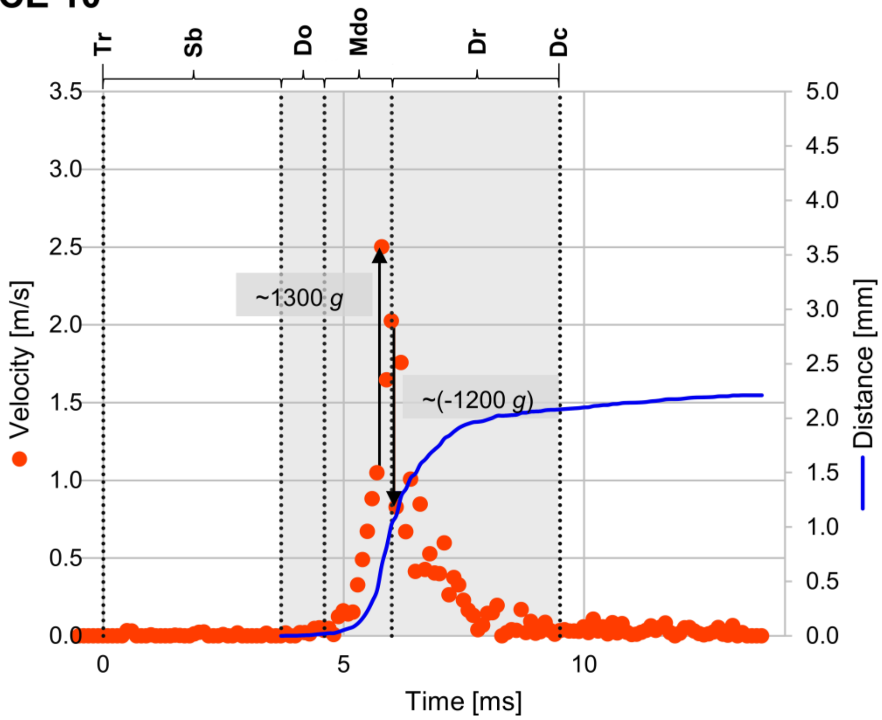

Figure S8: Interrelations of prey and trap movement steps and phases of CE 10.

CE 11

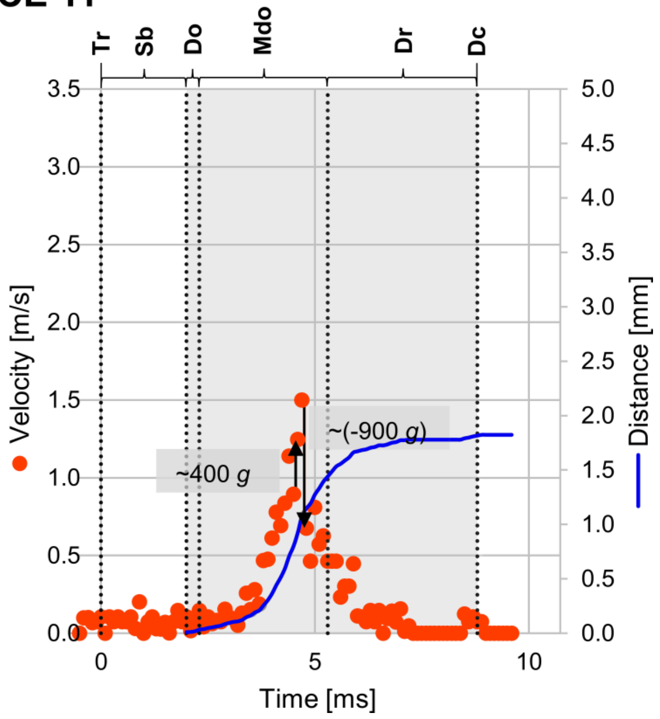

Figure S9: Interrelations of prey and trap movement steps and phases of CE 11.

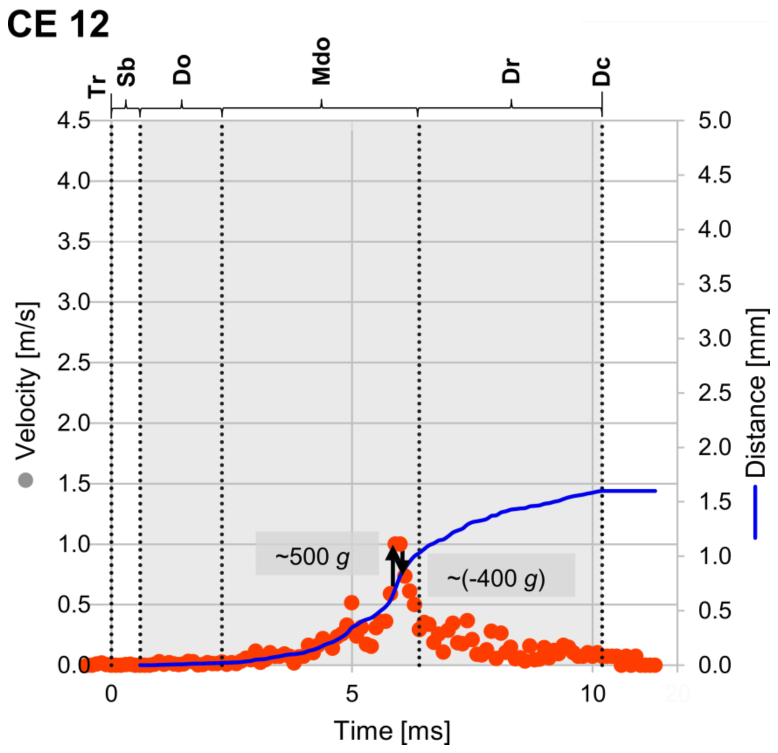

Figure S10: Interrelations of prey and trap movement steps and phases of CE 12.

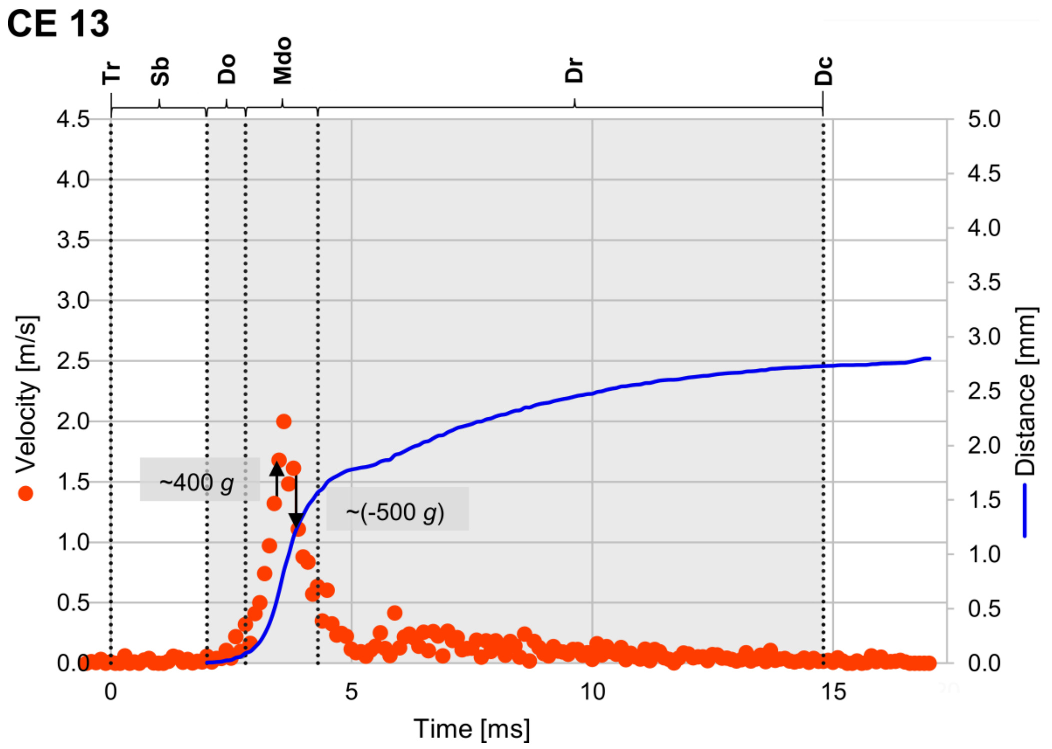

Figure S11: Interrelations of prey and trap movement steps and phases of CE 13.

CE 14

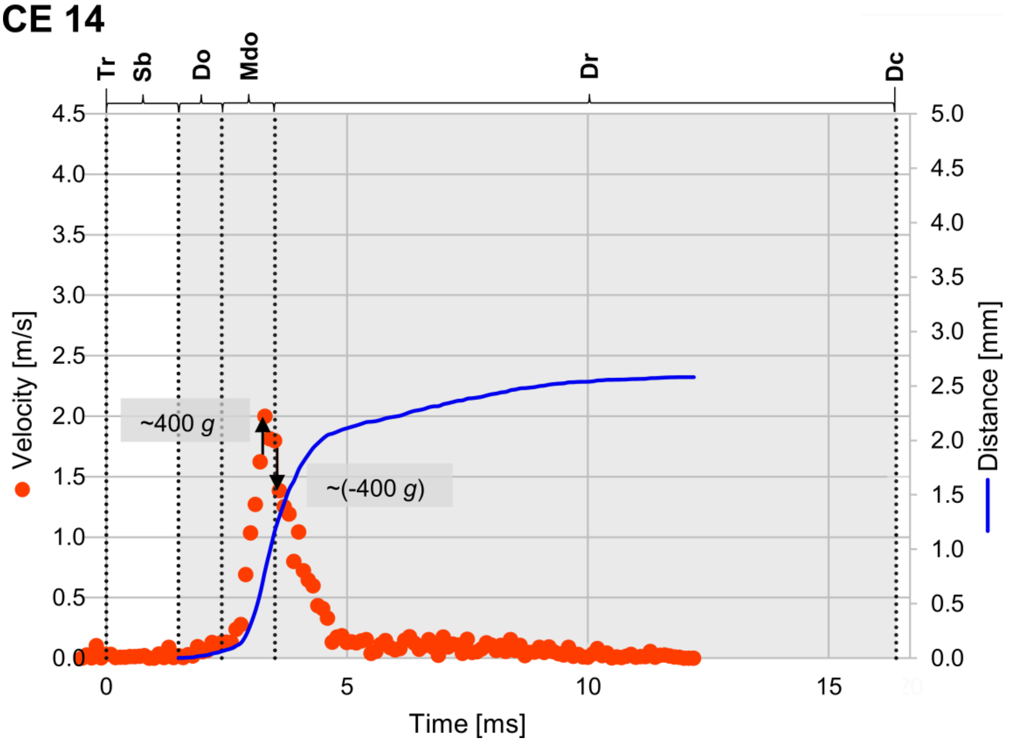

Figure S12: Interrelations of prey and trap movement steps and phases of CE 14.

Table S1: Prey organisms found in the 86 *U. australis* traps.

Taxon	Number of individuals found
Chydorus sphaericus	46
Ostracoda	13
Daphniidae	10
Ceriodaphnia dubia	8
Rotatoria	4
Insect larvae	2
Simocephalus vetulus	1
Eudiaptomus sp.	1

Legends for Movies S1-S15:

Movie S1: High-speed recording of prey capture event 1 (recording speed: 10.000 fps, playback rate: 10 fps) (.mov, 296x288 pixels, 296 KB).

Movie S2: High-speed recording of prey capture event 2 (recording speed: 10.000 fps, playback rate: 10 fps) (.mov, 320x308 pixels, 535 KB).

Movie S3: High-speed recording of prey capture event 3 (recording speed: 10.000 fps, playback rate: 10 fps) (.mov, 288x244 pixels, 214 KB).

Movie S4: High-speed recording of prey capture event 4 (recording speed: 10.000 fps, playback rate: 10 fps) (.mov, 320x308 pixels, 796 KB).

Movie S5: High-speed recording of prey capture event 5 (recording speed: 10.000 fps, playback rate: 10 fps) (.mov, 320x308 pixels, 588 KB).

Movie S6: High-speed recording of prey capture event 6 (recording speed: 10.000 fps, playback rate: 10 fps) (.mov, 320x320 pixels, 1.23 MB).

Movie S7: High-speed recording of prey capture event 7 (recording speed: 10.000 fps, playback rate: 10 fps) (.mov, 320x308 pixels, 1.04 MB).

Movie S8: High-speed recording of prey capture event 8 (recording speed: 10.000 fps, playback rate: 10 fps) (.mov, 288x272 pixels, 986 KB).

Movie S9: High-speed recording of prey capture event 9 (recording speed: 10.000 fps, playback rate: 10 fps) (.mov, 288x244 pixels, 255 KB).

Movie S10: High-speed recording of prey capture event 10 (recording speed: 10.000 fps, playback rate: 10 fps) (.mov, 320x308 pixels, 687 KB).

Movie S11: High-speed recording of prey capture event 11 (recording speed: 10.000 fps, playback rate: 10 fps) (.mov, 320x308 pixels, 503 KB).

Movie S12: High-speed recording of prey capture event 12 (recording speed: 10.000 fps, playback rate: 10 fps) (.mov, 288x244 pixels, 764 KB).

Movie S13: High-speed recording of prey capture event 13 (recording speed: 10.000 fps, playback rate: 10 fps) (.mov, 320x320 pixels, 572 KB).

Movie S14: High-speed recording of prey capture event 14 (recording speed: 10.000 fps, playback rate: 10 fps) (.mov, 320x3208 pixels, 644 KB).

Movie S15: High-speed recording of trigger hair deformation (recording speed: 10.000 fps, playback rate: 5 fps). The frames show details of the entrance region from Movie S10. The first part of the movie shows the process as originally filmed, the second part shows the same sequence with adjusted contrast and brightness for clarity. The process of trigger hair deformation is highlighted by an arrow (.mov, 160x123 pixels, 296KB).
